# Tenascin-X as a causal gene for classical-like Ehlers-Danlos syndrome

**DOI:** 10.3389/fgene.2023.1107787

**Published:** 2023-03-15

**Authors:** Emiko Okuda-Ashitaka, Ken-ichi Matsumoto

**Affiliations:** ^1^ Department of Biomedical Engineering, Osaka Institute of Technology, Osaka, Japan; ^2^ Department of Biosignaling and Radioisotope Experiment, Interdisciplinary Center for Science Research, Head Office for Research and Academic Information, Shimane University, Izumo, Japan

**Keywords:** tenascin-X, Ehlers-Danlos syndromes, clEDS, pain, tumor suppressor, fibrosis

## Abstract

Tenascin-X (TNX) is an extracellular matrix glycoprotein for which a deficiency results in a recessive form of classical-like Ehlers-Danlos syndrome (clEDS), a heritable connective tissue disorder with hyperextensible skin without atrophic scarring, joint hypermobility, and easy bruising. Notably, patients with clEDS also suffer from not only chronic joint pain and chronic myalgia but also neurological abnormalities such as peripheral paresthesia and axonal polyneuropathy with high frequency. By using TNX-deficient (*Tnxb* ^−/−^) mice, well-known as a model animal of clEDS, we recently showed that *Tnxb* ^−/−^ mice exhibit hypersensitivity to chemical stimuli and the development of mechanical allodynia due to the hypersensitization of myelinated A-fibers and activation of the spinal dorsal horn. Pain also occurs in other types of EDS. First, we review the underlying molecular mechanisms of pain in EDS, especially that in clEDS. In addition, the roles of TNX as a tumor suppressor protein in cancer progression have been reported. Recent *in silico* large-scale database analyses have shown that TNX is downregulated in various tumor tissues and that high expression of TNX in tumor cells has a good prognosis. We describe what is so far known about TNX as a tumor suppressor protein. Furthermore, some patients with clEDS show delayed wound healing. *Tnxb* ^−/−^ mice also exhibit impairment of epithelial wound healing in corneas. TNX is also involved in liver fibrosis. We address the molecular mechanism for the induction of *COL1A1* by the expression of both a peptide derived from the fibrinogen-related domain of TNX and integrin α11.

## Introduction

**FIGURE 1:**
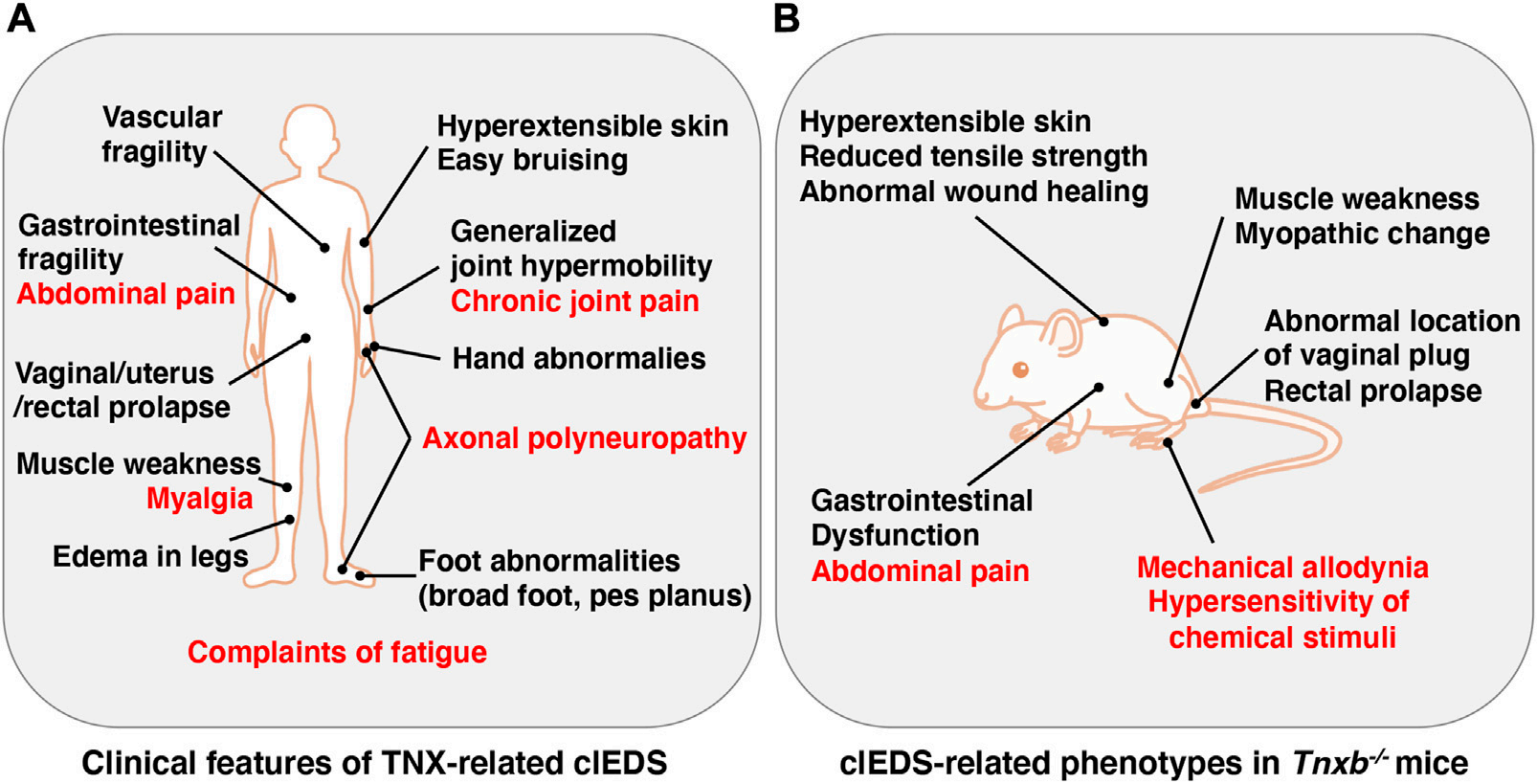
Clinical features of TNX*-*related clEDS **(A)** and clEDS-related phenotypes of *Tnxb* ^−/−^ mice **(B)**. Symptoms in many patients with TNX*-*related clEDS are shown in **(A)** (van Dijk et al., 2022). Major clEDS-related phenotypes exhibited in *Tnxb* ^−/−^ mice are shown in **(B)** (Matsumoto and Aoki, 2020). Complaints associated with pain are highlighted by red letters in **(A)** and **(B)**.

The Ehlers-Danlos syndromes (EDS) comprise a group of rare heritable connective tissue disorders mainly characterized by a variable degree of joint hypermobility, hyperextensible skin and fragility of connective tissues. Currently, 14 EDS are classified according to typical clinical features, and 20 causal genes that are mainly responsible for collagen and extracellular matrix (ECM) synthesis and maintenance have been identified (Malfait et al., 2020). Among the 14 types of EDS, non-collagenous classical-like EDS (clEDS) is the result of tenascin-X (TNX) deficiency with homozygous or compound heterozygous mutations in its gene (*TNXB*) (Burch et al., 1997; Schalkwijk et al., 2001; Malfait et al., 2017). The major clinical features of clEDS are generalized joint hypermobility, hyperextensible velvety skin without atrophic scarring, and easy bruising (Malfait et al., 2017) (Figure 1A).

A causal gene for clEDS, *TNXB*, was identified serendipitously as an opposite strand gene (*OSG*) with its 3′ genomic overlap with the steroid 21-hydroxylase gene (*CYP21A2*) in the human major histocompatibility complex (MHC) class III region (Morel et al., 1989). Further independent analyses of the MHC class III region revealed a novel gene having the highest homology with tenascin-C (TNC) and the *OSG* is the portion of the 3’ region of the gene, naming the novel gene *TNXB* (Matsumoto et al., 1992a; Matsumoto et al., 1992b; Bristow et al., 1993; Erickson, 1993). TNX is the largest glycoprotein in the tenascin family with a size of roughly 450 kDa and is composed of characteristic structural domains with a tenascin assemble region, heptad repeats, epidermal growth factor (EGF)-like repeats, fibronectin type III (FNIII)-like repeats, and a fibrinogen (FBG)-related domain (Bristow et al., 1993; Ikuta et al., 1998).

TNX is expressed prominently in a variety of tissues including the heart, skin, skeletal muscle, peripheral nerves, ligaments, tendons and the digestive tract, while there are very low expression levels in immune tissues such as the thymus, bone marrow and lymphocytes (Matsumoto et al., 1994; Geffrotin et al., 1995). Brain-derived neurotrophic factor (BDNF) has been identified as an up-regulator of TNX expression (Takeda et al., 2005) and glucocorticoids have been identified as a down-regulators of TNX expression (Sakai et al., 1996).

TNX has physiological functions in collagen deposition (Mao et al., 2002; Minamitani et al., 2004a), collagen stability (Mao and Bristow, 2001), physical property of collagen (Margaron et al., 2010) and collagen fibrillogenesis (Minamitani et al., 2004b; Egging et al., 2007). Several phenotypes tied to the function of TNX have been revealed by using *Tnxb* ^−/−^ mice (Matsumoto and Aoki, 2020) (Figure 1B).

In this review, we focus on the function of TNX in pain related to a characteristic of clEDS as well as in tumor suppression and fibrosis.

## Clinical characteristics of TNX-related clEDS

TNX-related clEDS was identified in 56 individuals from 44 families so far (van Dijk et al., 2022). The major clinical characteristics of TNX-related clEDS are skin hyperextensibility with velvety skin texture and absence of atrophic scaring (100% of patients), generalized joint hypermobility with or without recurrent dislocations (100%), and easy or spontaneous bruising of the skin including hematomas and ecchymoses (91%), as shown in Figure 1A (Malfait et al., 2017; van Dijk et al., 2022). It has been considered that the absence of atrophic scaring is a characteristic of clEDS, distinguish it from classical EDS, but mild atrophic scarring was observed in seven clEDS patients (Chen et al., 2016; Green et al., 2020). Additional musculoskeletal presentations of TNX-related clEDS are foot abnormalities including broad/plump forefoot, brachydactyly with excessive skin, pes planus, hallux valgus, and painful soles of the feet (81%), edema in the legs in the absence of cardiac failure (25%), hand anomalies (20%), and complaints of fatigue (53%) (van Dijk et al., 2022). Cardiovascular presentations of TNX-related clEDS are vascular fragility (27%), mild valvular abnormality (16%), and cardiomyopathy (5%) (van Dijk et al., 2022). Vascular fragility has been reported to cause major medical events such as rupture of the brachial vein and aneurysmal abdominal arteries (Demirdas et al., 2017; Micale et al., 2019). Neuromuscular presentations of TNX-related clEDS are subjective muscle weakness (37%), axonal polyneuropathy (14%), and atrophy of muscles in the hands and feet (4%) (van Dijk et al., 2022). Voermans et al*.* (2009) reported that TNX-deficient EDS patients show muscle weakness, myalgia, easy fatigability, and limited walking distance. Physical examination revealed mild-to-moderate muscle weakness, hypotonia, reduction of vibration sense, hyporeflexia, and impairment of mobility. Furthermore, clinical neurological studies showed axonal polyneuropathy and mild abnormal motor unit action potentials, and muscle ultrasound showed increased echo intensity and atrophy (Voermans et al., 2009). Interestingly, neuromuscular features have been observed in adults but not in children (Demirdas et al., 2017). Other presentations of TNX-related clEDS are gastrointestinal fragility including esophageal, small bowel and/or large bowel ruptures (16%), vaginal/uterus/rectal prolapse (21%), and other types of fragility including trachea rupture after intubation and defect of nasal cartilages after nose blowing (4%) (van Dijk et al., 2022).

## Pain in clEDS due to TNX deficiency

Pain is a common and severe symptom in patients with various types of EDS (Chopra et al., 2017; Syx et al., 2017; Malfait et al., 2021). Pain initially occurs as acute and localized musculoskeletal nociception in different joints and limbs in relation to hypermobility, subluxations, dislocations, soft-tissue injury, myalgias, and surgery (Chopra et al., 2017). However, pain related to EDS gradually becomes chronic (lasting for longer than 3 months) and assumes a more generalized distribution (Chopra et al., 2017; Syx et al., 2017). Among the various types of EDS, chronic pain is most frequent in hypermobile EDS. Chopra et al. (2017) reported that pain in patients with hypermobile EDS occurs in various forms including generalized body pain (incidence of 90%), soft-tissue pain (90%), dislocations (78%), and joint pain including pain in the shoulders (80%), hands (75%), knees (71%), temporomandibular joints (71%), spine (67%), and elbows (43%). In addition to musculoskeletal pain, patients with hypermobile EDS suffer from chronic fatigue (95%), neuropathic pain (68%), headaches (75%), gastrointestinal pain (86%), dysmenorrhea (73%), and vulvodynia/dyspareunia (42%). Pathological chronic pain is also caused by a lesion or disease of the somatosensory nervous system, and that pain is called neuropathic pain (Jensen et al., 2011). There is a high frequency of neuropathic pain in patients with EDS who have chronic pain (Chopra et al., 2017). Neuropathic pain occurs as spontaneous pain such as shooting, and burning, or stabbing pain, an increased response to normally noxious stimuli (hyperalgesia), and pain due to normally innocuous stimuli (allodynia) (Colloca et al., 2017).

**FIGURE 2:**
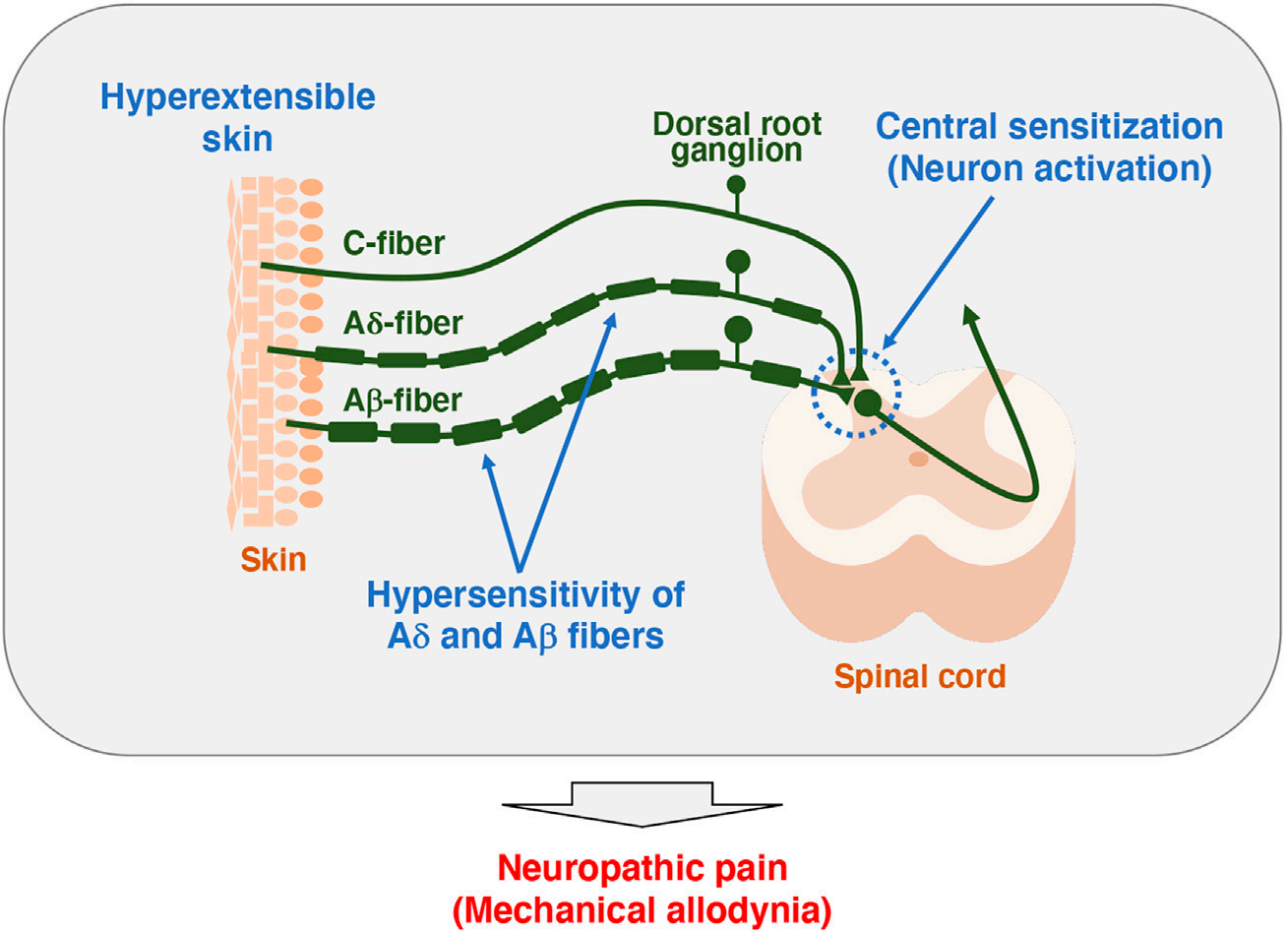
Model of pathogenesis for mechanical allodynia in *Tnxb* ^−/−^ mice. Somatosensory information is detected in the primary afferent fibers extending to the skin, which in turn is transmitted to the spinal cord and then to the brain. Unmyelinated C-fibers and lightly myelinated Aδ-fibers conduct noxious and thermal signals, whereas myelinated Aβ-fibers conduct innocuous signals such as touch and pressure (Moehring et al., 2018). *Tnxb* ^−/−^ mice exhibited increased sensitivity to innocuous mechanical stimuli but not thermal stimuli, indicating the induction of mechanical allodynia (Okuda-Ashitaka et al., 2020). Likewise, *Tnxb* ^−/−^ mice showed hypersensitization of myelinated Aδ- and Aβ-fibers but not C-fibers. Furthermore, levels of activated neuron markers, phosphorylation of extracellular signal-related kinase and NADPH-diaphorase activity of neuronal nitric oxide were increased in the spinal dorsal horn of *Tnxb* ^−/−^ mice compared to those in wild-type mice. Thus, TNX deficiency is involved in mechanical allodynia associated with hypersensitization of myelinated Aδ- and Aβ-fibers and central sensitization of the spinal cord.

It has been reported that TNX-related clEDS patients complain of chronic pain including joint pain, myalgia, back pain, abdominal pain, and fatigue (Figure 1A) (Schalkwijk et al., 2001; Voermans et al., 2009; Demirdas et al., 2017; Green et al., 2020; van Dijk et al., 2022). We first reported pain responses in a murine TNX-deficient EDS model (Okuda-Ashitaka et al., 2020). Our studies with *Tnxb* ^*−/−*^ mice showed increased sensitivity to innocuous mechanical stimuli but not to thermal stimuli such as cold and heat, suggesting that TNX deficiency is involved in the development of mechanical allodynia, a major feature of neuropathic pain (Figure 1B) (Okuda-Ashitaka et al., 2020). Furthermore, *Tnxb* ^*−/−*^ mice also exhibited hypersensitization of myelinated Aδ- and Aβ-fibers, but not unmyelinated C fibers, by using transcutaneous sine wave stimuli (Figure 2). TNX is highly expressed in tendons, ligaments, and peripheral nerves (Geffrotin et al., 1995). TNX exists in the perineurium, endoneurium, and Schwann cells in the sciatic nerve (Matsumoto et al., 2002; Sakai et al., 2017; Okuda-Ashitaka et al., 2020). Electron microscopy analysis of the sciatic nerve showed modestly smaller inner and outer diameters of myelinated fibers and reduced collagen fibril density in the endoneurium in *Tnxb* ^*−/−*^ mice (Voermans et al., 2011), whereas there was no significant difference in the numbers of axons or thickness of the myelin sheaths in *Tnxb* ^*−/−*^ mice (Matsumoto et al., 2002). Moderate changes of myelinated fibers and hypersensitization of myelinated Aδ- and Aβ-fibers in *Tnxb* ^*−/−*^ mice may be correlated to the axonal polyneuropathy in TNX-deficient EDS (Voermans et al., 2009). Axonal polyneuropathy is thought to be one of the mechanisms of neuropathic pain in EDS (Voermans et al., 2011). Furthermore, *Tnxb* ^*−/−*^ mice showed increased levels of anatomical neuronal activation markers, phosphorylated extracellular signal-regulated kinase and neuronal nitric oxide in the spinal dorsal horn, indicating that TNX deficiency induces spinal central sensitization, namely, another mechanism of neuropathic pain (Okuda-Ashitaka et al., 2020). Similar to pain responses in *Tnxb* ^*−/−*^ mice, a murine classical EDS model, type V collagen (COL5A1) haploinsufficient (*Col5a1* ^*+/−*^) mice, showed mechanical allodynia but not thermal hyperalgesia (Syx et al., 2020). *Col5a1* ^*+/−*^ mice showed a disorganization of Na_v_1.8-expressing fibers including above 90% C-fibers, with less fibers crossing the epidermis of footpad glabrous skin. These results indicated that pain in both TNX-related clEDS and COL5A1-related classical EDS corresponds to neuropathic pain associated with hypersensitization of myelinated Aδ- and Aβ-fibers, disorganization of Na_v_1.8-expressing fibers, and central sensitization of the spinal cord.

Additionally, TNX influences neuronal functions in gut tissues including abdominal pain (Figure 1A). *Tnxb* ^*−/−*^ mice show hypersensitivity of colonic nociceptive afferents and increased sensory neuron sprouting in the mucosa (Aktar et al., 2018). *Tnxb* ^*−/−*^ mice also exhibited gastric dysfunction associated with accelerated gastric emptying and hypersensitivity of gastric vagal mechanoreceptors (Figure 1B) (Aktar et al., 2019), which are consistent with TNX-related clEDS patients (Schalkwijk et al., 2001; Lindor and Bristow, 2005).

## TNX with tumor suppressive function

Previously, we demonstrated that *Tnxb* ^−/−^ mice bearing aggressive B16-BL6 melanoma cells exhibit promotion of tumor invasion and metastasis due to upregulation of matrix metalloproteinases *Mmp2* and *Mmp9* followed by enhanced activities of the MMPs (Matsumoto et al., 2001; Matsumoto et al., 2004). Conversely, overexpression of TNX in fibroblasts downregulated the expression of *Mmp2* (Matsumoto et al., 2004). In addition, silencing of long non-coding RNA (LncRNA) LINC01305 inhibited the progression of lung cancer by activating the TNX-mediated phosphatidylinositol 3-kinase (PI3K)/protein kinase B (Akt) signaling pathway (Yan et al., 2020). Moreover, it has been revealed that a functional variant in the *TNXB* promoter is associated with risk of esophageal squamous-cell carcinoma (ESCC) in the Chinese population, leading to the expression of *TNXB* being downregulated in ESCC tissues (Chang et al., 2018; Yang et al., 2020). Knockout of *TNXB* significantly increased cell proliferation of ESCC cells (Yang et al., 2020). These results suggest that TNX has a tumor suppressor role. Interestingly, when carcinoma cells were transplanted into the skin of nude mice, the expression of TNX was downregulated substantially not only in the transplanted tumor cells themselves but also in the surrounding tumor stroma (Sakai et al., 1996).

In conjunction with a tumor suppressor role of TNX, the expression of TNX was shown to be downregulated in most tumor tissues such as the lung, breast, prostate, colon, stomach, liver, kidney, skin melanoma, and leiomyoma by using *in silico* large database studies of the Gene Expression Omnibus (GEO) and The Cancer Genomic Atlas (TCGA) (Liot et al., 2020), although there are some discrepancies in the expression pattern of TNX in glioma and ovarian cancer compared with those of previous published data (Hasegawa et al., 1997; Kramer et al., 2015). In another study using the TCGA database for ECM gene dysregulation in cancer, 58 out of 249 ECM genes were identified as cancer-associated ECM genes and *TNXB* was found to be the most significantly downregulated among those genes in cancers (Chakravarthy et al., 2018). Even more interesting is that *TNXB* expression is inversely correlated with tumor progression and that a high level of TNX in tumor tissues predicts a good prognosis (Liot et al., 2020).

Meanwhile, as an exception, the expression of TNX is upregulated in malignant mesothelioma (Yuan et al., 2009; Davidson, 2011; Nakayama et al., 2019). This evidence suggests that TNX is applicable as a diagnostic marker of malignant mesothelioma since most other tumors are negative for TNX expression.

## Involvement of TNX in fibrosis and wound healing

Alcaraz et al. (2014) showed that a fibrinogen (FBG)-related domain of TNX (TNX-FBG) interacts with small latent TGF-β complex (SLC) and elicits the activation of its latent form into a bioactive form with integrin α11β1, leading to epithelial-to-mesenchymal transition in mammary epithelial cells. On the other hand, Liang et al. (2022) recently demonstrated that TNX-FBG interacts with mature TGF-β and impedes it from binding to its receptor, mediating flow-inducing suppression of endothelial-to-mesenchymal transition and atherosclerosis.

Previously, our group revealed that *Tnxb* ^−/−^ mice fed a high-fat and high-cholesterol diet with high levels of phosphorus and calcium (HFCD) exhibit less fibrotic characteristics in livers than those in wild-type mice, indicating the involvement of TNX in hepatic fibrosis (Yamaguchi et al., 2017). Fibrosis is a pathological sign of wound healing that replaces damaged tissue with collagen-rich scar tissue. We attempted to disclose the molecular mechanism by which TNX induces *in vitro* fibrosis such as the induction of type I collagen 1α (*COL1A1*) expression. Initially, we speculated that TGF-β and TNX-FBG with integrin α11β1 are involved in the induction of *COL1A1* expression since interaction of the TNX-FBG domain and TGF-β was reported previously (Alcaraz et al., 2014) and TGF-β is a well-known central mediator of fibrosis (Dewidar et al., 2019). However, contrary to our initial expectation, we found that the Yes-associated protein 1 (YAP1) signaling pathway through integrin α11β1 plays a major role in the induction of *COL1A1* expression by expression of the TNX-FBG domain in human hepatic stellate LX-2 cells and that the minimum 15-amino acid (aa) sequence derived from the TNX-FBG domain is required for the induction of *COL1A1* expression in the LX-2 cells (Matsumoto et al., 2022). Since integrin α11β1 is known to be a receptor for type I collagen (COL1) and type II collagen (Zhang et al., 2003), it is yet to be determined whether interaction of the TNX-FBG domain with integrin α11β1 is direct or indirect for the induction of *COL1A1*expression.

According to previous reports, 41% of patients with TNX-deficient clEDS showed delayed wound healing (Demirdas et al., 2017). Notably, the corneas of *Tnxb* ^−/−^ mice that underwent epithelium debridement exhibited impairment of epithelial wound healing due to increased neutrophil infiltration and activation of reactive oxygen species (Sumioka et al., 2021). TNX might also be involved in the angiogenetic process during wound healing. Injury-induced corneal stromal angiogenesis in *Tnxb* ^−/−^ mice was impaired (Sumioka et al., 2018).

## Conclusion and perspectives

In this review, we described the molecular mechanisms of pain caused by TNX deficiency as well as by mutation of collagens mimicking the characteristics of EDS, the function of TNX as a tumor suppressor, and the involvement of TNX in fibrosis.

Concerning pain associated with malfunction of the ECM, the contribution of TNX-deficient clEDS and COL5A1 haploinsufficiency-related classical EDS to the development of neuropathic pain has been revealed by using a murine EDS model. Patients with EDS take large amounts of medications such as acetaminophen, non-steroid anti-inflammatory drugs (NSAIDs), anticonvulsants, antidepressants, opioids, and lidocaine; however, current managements are inadequate (Demes et al., 2020; Whalen and Crone, 2022). Interestingly, mechanical allodynia in *Tnxb* ^*−/−*^ mice was inhibited by the anticonvulsant drug gabapentin and the mu-opioid agonist [D-Ala^2^, N-MePhe^4^, Gly-ol^5^]-enkephalin (DAMGO) but not by the NSAID indomethacin (Okuda-Ashitaka et al., 2020). In the future, more efficacious approaches in line with the mechanisms causing pain in patients with EDS are expected.

Concerning tumor progression associated with TNX expression, the increased expression of TNX in malignant mesothelioma is very interesting, despite its expression being downregulated in most tumor tissues. Yuan et al. (2009) showed some splice variants of TNX are observed in malignant mesothelioma. The splice variants of TNX might be involved in the malignancy of mesothelioma. In the future, analyses of not only splice variants of TNX itself but also proteins that interact with their splice variants are needed to reveal the specific function of TNX in malignant mesothelioma.

Finally, we showed that *COL1A1* expression was induced by expression of both the 15-aa peptide in the TNX-FBG domain and integrin α11 in hepatic stellate LX-2 cells *in vitro* (Matsumoto et al., 2022). Further experiments are needed to determine whether expression of the 15-aa peptide from the TNX-FBG domain in liver can induce *COL1A1* expression leading to hepatic fibrosis *in vivo*.
